# Clinical Nurses' Attitudes and Self‐Reported Practices of Family Nursing in Japan Following COVID‐19 Visitation Restrictions: A Cross‐Sectional Study

**DOI:** 10.1111/jocn.70181

**Published:** 2025-12-11

**Authors:** Makoto Tsukuda, Junko Honda, Keisuke Nojima, Yoshiyasu Ito, Hiromi Asada

**Affiliations:** ^1^ Faculty of Nursing Hyogo Medical University Kobe Japan; ^2^ Research Institute of Nursing Care for People and Community University of Hyogo Akashi Japan; ^3^ Department of Nursing Kyoto Tachibana University Kyoto Japan; ^4^ Faculty of Nursing Science Tsuruga Nursing University Tsuruga Japan; ^5^ College of Nursing Art and Science University of Hyogo Akashi Japan

**Keywords:** COVID‐19, family systems theory, FINC‐NA, visitation restrictions

## Abstract

**Aim:**

To examine clinical nurses' attitudes towards and self‐reported experiences of family nursing in Japan following the relaxation of COVID‐19 visitation restrictions. Particular attention is paid to early career nurses whose formative training occurred during visitation bans. The study focused on nurses' negative perceptions and emotional burdens associated with family involvement.

**Design:**

A quantitative‐dominant mixed‐methods cross‐sectional study reported in accordance with the STROBE guideline.

**Methods:**

Using a convenience sampling approach, a self‐administered, paper‐based questionnaire was distributed to clinical nurses in four general hospitals in Japan between January and May 2024. The questionnaire consisted of four parts: demographic and professional background, learning methods related to family nursing, 17 items including negatively valenced statements adapted from the *Families' Importance in Nursing Care–Nurses' Attitudes* (FINC‐NA) scale, and one open‐ended question. Quantitative data were analysed using descriptive statistics and *t*‐tests, and qualitative responses were thematically analysed.

**Results:**

Of 1921 nurses invited, 957 responded (response rate: 49.8%), and data from 892 valid responses were analysed. Overall, the nurses demonstrated positive recognition of family nursing as a professional value but also reported lingering emotional burdens and practical challenges when interacting with families. Early‐career nurses who began practice during the pandemic showed greater uncertainty and lower affective engagement. Thematic analysis revealed five key themes: relational disruption, emotional stress, moral conflict, reappraisal of family engagement and ongoing barriers.

**Conclusion:**

The findings underscore the need to structurally and educationally reintegrate families into nursing care. Simulation‐based training, clear institutional policies and hybrid communication models are essential to rebuild relational continuity and support nurses' emotional and ethical capacity for family nursing.

**Implications for the Profession and/or Patient Care:**

The findings highlight the need to structurally and educationally reintegrate families into clinical care to address the emotional burden and ambivalence reported by nurses. Organisational support—such as clear visitation policies, simulation‐based education and reflective opportunities—can help rebuild nurses' relational competence and confidence in engaging with families. Creating supportive learning environments, including on‐the‐job mentoring and team‐based reflection, may further facilitate the restoration of family nursing.

**Impact:**

This study addressed how prolonged COVID‐19 visitation restrictions disrupted family nursing practice in Japan, created generational differences in nurses' competencies, and shaped nurses' perceptions of family involvement. Nurses reported emotional strain, feelings of being monitored and lack of time when families were present. Early career nurses showed lower relational engagement, while experienced nurses expressed moral distress. ‘Latent indifference’ was also noted. The findings provide valuable insights for healthcare organisations, nurse educators and policymakers by informing strategies to reintegrate families into patient care, improve discharge planning and strengthen training models.

**Reporting Method:**

The STROBE checklist.

**Patient or Public Contribution:**

No patient or public contribution.

## What Does This Paper Contribute to the Wider Global Clinical Community?

- This study demonstrates how prolonged COVID‐19 visitation restrictions reshaped nurses' perceptions of family involvement in patient care, revealing emotional burden, relational disruption and generational differences in competencies.
- It identifies the risk of ‘latent indifference’, whereby the absence of direct engagement with families weakens nurses' long‐term involvement in family nursing.
- It provides evidence‐based recommendations—including culturally tailored simulation training, explicit policies, and hybrid communication models—to reestablish sustainable family nursing practice.

## 1 Introduction

Family nursing is a foundational component of holistic care, which recognises the family not as a peripheral presence but as an integral unit in the care process. Rooted in family systems theory (Wright and Leahey 2013), this perspective emphasises that the health and well‐being of one family member is closely connected to those of the others. Thus, nurses are expected to assess family dynamics, respond to collective needs and actively involve families as partners in care delivery.

In Japan, direct interaction with patients' families has traditionally been a core component of nursing practice, reflecting culturally embedded values, such as harmony, collective responsibility and filial piety. These cultural expectations have shaped nurses' attitudes and relational competencies, particularly in end‐of‐life and institutional care settings (Fukui et al. 2021; Moriyama 2008). However, the onset of the COVID‐19 pandemic in 2020 led to strict visitation restrictions, with hospitals enforcing near‐total bans on family visits (Krewulak et al. 2024; Moss et al. 2021). These restrictions significantly reduced opportunities for nurses to communicate with patients' families, coordinate care and build trust.

This disruption was particularly consequential for nurses who entered clinical practice during this period. Without direct interactions at the bedside, these nurses were deprived of the formative experiences necessary to develop family nursing values and competencies (Dentice et al. 2024). By contrast, more experienced nurses who had worked in family‐inclusive care settings before the pandemic reported moral distress owing to the absence of family participation (Carter et al. 2021). This generational disparity in exposure and expectations has raised concerns among educators and leaders about the long‐term implications for nurses' competencies in and attitudes towards family engagement (Dentice et al. 2024).

By mid‐2023, most Japanese hospitals had begun easing their visitation restrictions. However, the nearly 3‐year absence of family presence may have contributed to differences in how nurses perceived and implemented family nursing, depending on their training period and clinical environment. Understanding these differences is essential for reestablishing sustainable family nursing practices.

## 2 Background

### 2.1 Measurement Context and Critical Perspective

The Families' Importance in Nursing Care–Nurses' Attitudes (FINC‐NA) scale (Benzein et al. 2008) has been widely used to assess nurses' attitudes towards family involvement in clinical care. Its four subscales (Family as a Resource in Nursing Care [Fam‐RNC], Family as a Conversational Partner [Fam‐CP], Family as a Burden [Fam‐B] and Family as its Own Resource [Fam‐OR]) offer a comprehensive framework for evaluating professional perceptions of family nursing.

However, recent scholarship has questioned the scale's capacity to fully capture nurses' clinical behaviour, particularly under high pressure or crisis conditions, such as the COVID‐19 pandemic. During this period, nurses who scored high on the FINC‐NA often faced barriers that prevented them from implementing family engagement, including strict infection control protocols, increased workloads and ethical uncertainty (Shamali et al. 2023). While many endorsed the theoretical importance of family participation, during this period, few nurses could include families in care planning or communication processes. This disconnect highlights the potential gap between attitudinal endorsement and behavioural feasibility, especially in the absence of structural support.

Considering these concerns, this study focused on negatively valenced FINC‐NA items to better detect latent discomfort, operational burdens and attitudinal shifts that may not be captured by positively framed questions, particularly in high‐context cultures like Japan. This methodological choice was based on the hypothesis that lifting the visitation restrictions might have inadvertently increased nurses' perceived burden, emotional fatigue and operational complexity, particularly among early career nurses with limited prior exposure to family interactions. As families re‐entered the clinical environment after years of forced separation, their presence may have added to the stress of the already strained care systems.

Furthermore, in high‐context cultures like Japan, where nonverbal communication, relational harmony, and implicit role expectations are highly institutionalised (Fukui et al. 2021), attitudinal scales such as the FINC‐NA may overrepresent aspirational norms while failing to detect situational disengagement or context‐bound ambivalence. In such settings, passive withdrawal from family engagement may not signify active resistance but rather reflect institutional invisibility or a lack of structured exposure.

Importantly, the FINC‐NA does not account for structural constraints such as insufficient time, space, staffing or training opportunities. Even when nurses have positive attitudes, the absence of organisational reinforcement may prevent meaningful implementation. Therefore, interpretations of the FINC‐NA scores, particularly in post‐pandemic contexts, must be critically situated within the structural and cultural realities that shape everyday clinical practice. In Japan's high‐context communication culture, relational harmony and implicit role expectations often shape interactions with families. These cultural features can obscure signs of disengagement or discomfort, underscoring the need for instruments sensitive to subtle shifts in attitude.

### 2.2 Theoretical Framework: Family Systems Theory

Family systems theory conceptualises the family as a dynamic and interdependent unit in which the health and behaviour of each member influence the functioning of the entire system. In nursing, this theory encourages attention not only to individual patient needs but also to relational, emotional and contextual factors within the family unit.

The COVID‐19‐related visitation restrictions significantly limited nurses' ability to observe or interact with family members, include them in care processes, and assess family needs. These constraints created structural barriers that disrupted the relational foundation of family nursing and may have altered how nurses now perceive families' role in care. From a family systems theory perspective, this disruption represents not merely a logistical inconvenience but a breakdown of the therapeutic triangle linking patients, families and nurses, reducing nurses' capacity to recognise and engage in systemic interdependence.

## 3 The Study

This study investigated how nurses' perceptions of family nursing have been shaped by the COVID‐19 pandemic, particularly in relation to the suspension and subsequent reintroduction of family visitation. By comparing nurses with different lengths of clinical experience working across different clinical settings, this study sought to:

- Examine how COVID‐19 visitation restrictions influenced nurses' perceptions of family involvement in care.
- Explore how demographic and professional characteristics, especially clinical experience and care settings, contribute to these perceptions.

By clarifying how structural disruptions influenced nurses' views and attitudes, this study contributes to the development of evidence‐based strategies for reestablishing and sustaining family‐centred care in the post‐pandemic healthcare environment. In addition, it provides culturally grounded insights into how relational norms and professional values can be reconstructed in the face of crisis‐driven policy shifts.

## 4 Methods

### 4.1 Study Design

A quantitative‐dominant mixed‐methods cross‐sectional design was employed to investigate clinical nurses' attitudes and self‐reported practices of family nursing practice in the context of the post‐COVID‐19 visitation policy relaxation in Japan. This study explores how prolonged visitation restrictions influenced nurses' attitudes towards and engagement in family nursing by comparing responses across different levels of clinical experience and care settings. Data were collected between January and May 2024 using a structured self‐administered questionnaire specifically developed for this study. While the primary analysis was quantitative, supplementary qualitative content analysis of open‐ended responses was conducted to provide contextual understanding and to complement the quantitative findings. The study was reported in accordance with the Strengthening the Reporting of Observational Studies in Epidemiology (STROBE) guideline for cross‐sectional studies (von Elm et al. 2007). The completed STROBE checklist is provided in Appendix S1.

### 4.2 Participants

The participants were clinical nurses employed at four general hospitals in various regions of Japan. A total of 1921 nurses were invited to participate. The eligibility criteria included involvement in direct patient care across a range of departments, including general medical and surgical wards, intensive care units, paediatric wards and emergency departments. Participation was voluntary and anonymous. Paper‐based surveys were distributed and collected through the internal administrative systems of each hospital. These four general hospitals were selected because they admitted COVID‐19 patients during the pandemic, enforced strict visitation bans for families, and lifted those restrictions in May 2023. Although minor variations existed in the timing and details of visitation policy relaxation among the four hospitals, all had lifted their restrictions by May 2023 and permitted limited in‐person visits under similar conditions (e.g., allowing only one family member per patient for approximately 10 min visit between 14:00 and 16:00). Thus, the overall visitation context during the data collection period was comparable across sites. The sample size reflected the total number of eligible clinical nurses employed at these hospitals at the time of data collection. No a priori sample size calculation was performed because this was an exploratory cross‐sectional study using a convenience sample of available clinical nurses.

### 4.3 Instruments

Data were collected using a structured self‐administered questionnaire designed for this study. The questionnaire comprised four main sections described in the following subsections.

#### 4.3.1 Demographic and Professional Background

Participants provided information on gender, age, educational background, professional qualifications (e.g., registered nurse, midwife, certified nurse, certified nurse specialist), years of clinical experience, current department and job title. They were also asked how they had learned about family nursing (e.g., through on‐the‐job training, mentorship, peer discussions, in‐hospital workshops, or external training programs).

For analytical purposes, years of clinical experience were categorised to reflect potential differences in exposure to family presence before, during, and after the COVID‐19 pandemic. Nurses with 1–3 years of experience likely began their careers during the period of restricted family visitation and may have encountered in‐person family engagement only after the formal relaxation of policies in May 2023. By contrast, nurses with four or more years of experience were more likely to have practiced in clinical settings before the pandemic, when direct interaction with patients' families was routine. These experience‐based distinctions were used to explore whether the timing of nurses' professional entry influenced their perceptions of family nursing.

#### 4.3.2 Attitudes and Perceived Practices of Family Nursing

Seventeen items were constructed to assess the nurses' perceptions of family nursing, focusing on the transitional period during which visitation restrictions were lifted. Item development was guided by the FINC‐NA (Benzein et al. 2008; Saveman et al. 2011), a psychometrically validated scale commonly used to measure nurses' attitudes towards family involvement in care. The original scale consists of 26 items across its four subscales (Fam‐RNC, Fam‐CP, Fam‐B and Fam‐OR) and has demonstrated robust internal consistency (Cronbach's α = 0.73–0.92) and test–retest reliability.

For this study, 14 items were adapted from the FINC‐NA: 10 from the Fam‐RNC subscale and four from the Fam‐B subscale. These items were translated and culturally adapted into Japanese using forward–backward translation by bilingual experts. Wording adjustments were made to reflect the COVID‐19 visitation context, and three original items were added based on pre‐testing with 10 clinical nurses to ensure clarity and relevance. An additional three original items were developed to reflect evolving attitudes towards family presence during the relaxation of the COVID‐19 restrictions. All items were rated on a 6‐point Likert‐type scale ranging from 1 (*strongly disagree*) to 6 (*strongly agree*). The internal consistency of the 17 items was evaluated using Cronbach's alpha after data collection to confirm the reliability of the adapted instrument. The total scale score was not computed, as each item was analysed independently to examine specific aspects of nurses' perceptions.

#### 4.3.3 Current Perceptions and Experiences of Family Support

To explore nurses' perceived impact of visitation restrictions on their family nursing practice, participants were asked an open‐ended question:

‘Please describe your current thoughts or experiences regarding how you support patients' families in your clinical practice’. The open‐ended responses were analysed thematically using Braun and Clarke's six‐step approach. Two researchers independently coded the data, reconciled coding discrepancies through discussion, and refined the codebook iteratively. Themes were derived inductively, with representative quotations selected to illustrate key findings. To ensure the qualitative analysis' rigour and trustworthiness, reflexivity was maintained through regular team discussions in which researchers examined their assumptions and potential biases.

The two analysts (one clinical nurse researcher experienced in family nursing and one qualitative research specialist) independently conducted the coding, compared interpretations and reached consensus through discussion. The researchers had no supervisory or hierarchical relationship with the participants, minimising the risk of response bias.

### 4.4 Data Analysis

Quantitative data were analysed using SPSS version 28. Descriptive statistics (means, standard deviations and frequencies) were calculated for demographic variables and item responses. Because the study yielded a large sample (*N* = 892), the data were considered approximately normally distributed according to the central limit theorem, and parametric tests were applied. Group differences in perceptions were assessed using independent sample *t*‐tests. Because the comparison involved only two groups, post hoc tests were not required. The open‐ended responses were analysed thematically to identify recurring patterns and concerns.

### 4.5 Ethical Considerations

This study was approved by the Institutional Ethics Review Board of the University of Hyogo (Approval No. 2023F24; Date: 22 November 2023). The study was conducted in accordance with the Declaration of Helsinki and institutional regulations. Participation was voluntary and anonymous; no personally identifiable information was collected. For the paper‐based survey, informed consent was obtained in writing. Participants received an information sheet explaining the study purpose, procedures, confidentiality and their right to withdraw at any time.

Submission of the completed anonymous questionnaire was considered to imply consent to participate. Where applicable, administrative permission was obtained from participating hospitals prior to data collection.

## 5 Results

### 5.1 Participant Characteristics

Of the 1921 nurses invited to participate, 957 returned the questionnaire (response rate: 49.8%). After excluding incomplete responses, 892 valid responses were included in the final analysis. Table 1 summarises the participants' demographic and professional characteristics. Most respondents were female (92.0%) and had over 11 years of clinical experience (40.9%). Most participants were employed in general wards (56.8%), with smaller proportions working in acute care units (13.8%), outpatient departments (13.1%), or paediatric/perinatal wards (4.6%). Incomplete responses were excluded if more than 20% of items were missing, resulting in the exclusion of 65 cases (6.8%). To examine the potential for bias, basic demographic variables (age, gender, years of experience and department) were compared between included and excluded respondents, and no significant differences were found. Therefore, the exclusion of incomplete responses was unlikely to have introduced systematic bias. The distribution of respondents across units is presented in Table 1 to clarify potential variations by work setting.

**TABLE 1:** Nurse attributes (*N* = 892).

Characteristics	Categories	*n* (%)
Age	21–24	188 (21.1)
	25–29	214 (24.0)
	30–40	205 (23.6)
	> 40	263 (29.5)
Gender	Male	75 (8.4)
	Female	812 (91.0)
	Other	3 (0.3)
Educational background	Vocational school	535 (59.9)
	University	334 (37.4)
	Graduate school	12 (1.3)
Clinical experience (years)	< 1	71 (8.0)
	2–3	162 (18.2)
	4–5	105 (11.8)
	6–10	171 (19.2)
	> 11	365 (40.9)
Affiliation	General wards	507 (56.8)
	Acute care units	123 (13.8)
	Paediatric/perinatal wards	41 (4.6)
	Outpatient departments	117 (13.1)
Position	Nurse manager	15 (1.7)
	Assistant nurse manager	101 (11.3)
	General nurse	771 (86.4)

### 5.2 Learning Methods Related to Family Nursing

Table 2 summarises how nurses reported learning about family nursing. The most common source was daily nursing practice (78.0%), followed by peer conferences or case discussions (58.6%) and guidance from senior staff or role models (45.3%). Less frequently reported methods included in‐hospital training or workshops (26.8%), peer‐led study sessions (15.5%) and off‐site or external training/workshops (12.0%). Only a small proportion (2.2%) reported other methods. Respondents were permitted to select multiple options, resulting in a total percentage exceeding 100%.

**TABLE 2:** Learning methods related to family nursing (*N* = 892).

Items	*n* (%)
1. Daily nursing practice	696 (78.0)
2. Guidance from senior staff or role models	404 (45.3)
3. Peer conferences or case discussions	523 (58.6)
4. Peer‐led study sessions	138 (15.5)
5. In‐hospital training or workshops	239 (26.8)
6. Off‐site or external training/workshops	107 (12.0)
7. Others	20 (2.2)

*Note:* Respondents were permitted to select multiple options. Therefore, the total percentage exceeds 100%.

### 5.3 Perceptions of Family Nursing Practice

#### 5.3.1 Internal Consistency of the Instrument

The 17‐item scale used in this study, which was developed and culturally adapted from the FINC‐NA scale as described in the Methods section, demonstrated acceptable internal consistency, with a Cronbach's alpha of 0.795. Although Cronbach's alpha was calculated as a reference for internal consistency, total scale scores were not computed because each item was analysed independently to examine specific aspects of nurses' attitudes towards family involvement in the post‐restriction context.

#### 5.3.2 General Attitudes Towards Family Involvement

Table 3 presents descriptive statistics for each item. Overall, nurses demonstrated generally positive attitudes towards family engagement. For instance, a high proportion of nurses agreed with the statement that building good relationships with families enhances their job satisfaction (Item 5). Likewise, many supported the active participation of family members in patient care (Item 4). In addition, the vast majority acknowledged that family visitation is important for both patients and their families (Items 12 and 13) and expressed positive feelings about the resuming of visits (Item 10). These results suggest that, despite the prolonged visitation restrictions during the pandemic, nurses have retained a strong recognition of family nursing as a core professional value. Nevertheless, some nurses expressed mixed feelings about family involvement, suggesting that emotional and practical tensions persist in balancing relational care with the demands of clinical efficiency.

**TABLE 3:** Perception of family nursing practice (*N* = 892).

Items	Completely disagree *n* (%)	Mostly disagree *n* (%)	Somewhat disagree *n* (%)	Somewhat agree *n* (%)	Mostly agree *n* (%)	Completely agree *n* (%)
1. The presence of family members eases my workload	15 (1.7)	44 (4.9)	191 (21.4)	318 (35.7)	234 (26.2)	88 (9.9)
2. The presence of family members gives me a feeling of security	14 (1.6)	42 (4.7)	194 (21.7)	320 (35.9)	232 (26.0)	89 (10.0)
3. The presence of family members is important to me as a nurse	2 (0.2)	20 (2.2)	60 (6.7)	349 (39.1)	340 (38.1)	118 (13.2)
4. Family members should be invited to actively take part in the patient's nursing care	0 (0.0)	18 (2.0)	90 (10.1)	336 (37.7)	322 (36.1)	124 (13.9)
5. A good relationship with family members gives me job satisfaction	8 (0.9)	25 (2.8)	115 (12.9)	365 (40.9)	289 (32.4)	90 (10.1)
6. Getting involved with families gives me a feeling of being useful	6 (0.7)	35 (3.9)	175 (19.6)	393 (44.1)	219 (24.6)	64 (7.2)
7. I gain a lot of worthwhile knowledge from families which I can use in my work	3 (0.3)	12 (1.3)	95 (10.7)	386 (43.3)	315 (35.3)	81 (9.1)
8. The presence of family members is important for the family members themselves	0 (0.0)	6 (0.7)	25 (2.8)	169 (18.9)	345 (38.7)	346 (38.8)
9. It is important to spend time with families	0 (0.0)	7 (0.8)	18 (2.0)	151 (16.9)	349 (39.1)	367 (41.1)
10. I'm glad that family visitation has resumed	1 (0.1)	12 (1.3)	42 (4.7)	190 (21.3)	317 (35.5)	329 (36.9)
11. Family visitation is not necessary	493 (56.3)	199 (22.3)	102 (11.4)	52 (5.8)	33 (3.7)	12 (1.3)
12. Family visitation is important to the patient	2 (0.2)	9 (1.0)	22 (2.5)	131 (14.7)	306 (34.3)	421 (47.2)
13. Family visits to the patient are important to the family	1 (0.1)	7 (0.8)	20 (2.2)	134 (15.0)	321 (36.0)	401 (45.0)
14. The presence of family members makes me feel that they are checking up on me	43 (4.8)	110 (12.3)	186 (20.9)	331 (37.1)	167 (18.7)	55 (6.2)
15. The presence of family members makes me feel stressed	96 (10.8)	197 (22.1)	344 (38.6)	188 (21.1)	51 (5.7)	14 (1.6)
16. The presence of family members holds me back in my work	99 (11.1)	213 (23.9)	318 (35.7)	207 (23.2)	44 (4.9)	10 (1.1)
17. I don't have time to take care of families	40 (4.5)	124 (13.9)	188 (21.1)	369 (41.4)	138 (15.5)	32 (3.6)

#### 5.3.3 Ambivalence and Emotional Burden

Several items revealed hesitation or perceived burden associated with family presence. For example, fewer nurses agreed that family presence eased their workload (Item 1), and many reported uncertainty or emotional strain when families were nearby (Item 2, 14 and 15). Such responses illustrate an enduring ambivalence: nurses value family inclusion but also perceive it as a potential source of stress, surveillance or disruption.

This emotional burden may reflect residual effects of pandemic‐related restrictions and heightened vigilance, which disrupted relational routines and altered expectations for family interaction. The findings highlight a persistent gap between the ideal of family nursing and clinical practice's operational realities, emphasising the need for institutional environments that support both patient–family engagement and nurses' emotional well‐being.

#### 5.3.4 Perceived Burden by Experience and Department

Table 4 presents the comparison of negative perception scores between nurses with 2–3 years of experience (who started clinical practice during the COVID‐19 visitation restrictions) and those with ≥ 4 years of experience (who started before the pandemic).

**TABLE 4:** Comparison of negative perception items between nurses with 2–3 years and ≥ 4 years of clinical experience (*N* = 803).

Item	*n* (2–3 years)	Mean (SD) 2–3 years	*n* (≥ 4 years)	Mean (SD) ≥ 4 years	Mean diff (2–3 years−≥ 4 years)	Hedges' *g* [95% CI]	*t* (Welch, df)	*p*	*q*
Family visitation is not necessary	162	2.09 (1.355)	641	1.77 (1.147)	0.33	0.27 [0.10, 0.45]	2.81 (222.7)	**0.005**	**0.027**
Family presence makes me feel checked	162	3.86 (1.259)	641	3.68 (1.215)	0.18	0.15 [−0.03, 0.32]	1.62 (242.4)	0.106	0.266
Family presence makes me feel stressed	162	3.07 (1.132)	641	2.94 (1.094)	0.13	0.12 [−0.05, 0.29]	1.36 (242.6)	0.176	0.293
Family presence holds me back at work	162	3.01 (1.153)	641	2.90 (1.081)	0.11	0.10 [−0.08, 0.27]	1.07 (237.5)	0.287	0.359
I don't have time to care for families	162	3.55 (1.089)	641	3.62 (1.164)	−0.07	−0.06 [−0.23, 0.11]	−0.70 (261.9)	0.482	0.482

*Note:* Independent‐sample comparisons were conducted between nurses with 2–3 years and ≥ 4 years of experience. Items were rated on a 1–6 scale (higher = more negative). Group differences tested with Welch's *t*; effect size = Hedges' *g* (95% CI). Multiple testing controlled by Benjamini–Hochberg FDR at *q* = 0.05. Bold values indicate *q* < 0.05.

After controlling for multiple testing, no significant differences were observed across items, although early‐career nurses showed slightly higher mean scores on several items, particularly regarding the necessity of family visitation and feelings of being monitored.

These findings suggest that negative perceptions towards family involvement are not limited to early‐career nurses but are shared across experience levels, indicating the need for organisational‐level interventions rather than individual‐level training alone.

### 5.4 Qualitative Findings: Impact of Visitation Restrictions on Practice

Of the 892 respondents, 152 (17.0%) provided written responses to the open‐ended question. A thematic analysis of the open‐ended responses revealed five prominent categories (Table 5), each of which is discussed in the following subsections.

**TABLE 5:** Thematic analysis of nurses' perspectives on family nursing under visitation restrictions.

Themes	Subthemes
Disruption of family nursing	I have less time to interact with family members It is difficult to gather information directly from families Relationships with families have weakened, and trust has been compromised There are challenges in young nurses' ability to provide family nursing (e.g., assisting with hygiene and environmental adjustments) Opportunities to practice family nursing have decreased, leading to skill deterioration I feel sorry that I don't have time to engage with families There has been an increase in phone inquiries from families about the patient's condition It is difficult to find effective ways to communicate with both patients and families to help them feel at ease Discrepancies have emerged between families' perceptions and the actual condition of patients It has become more difficult to visualise or plan for patients' post‐discharge lives Families tend to make excessive demands when they are unable to visit
Emotional stress and moral conflict	I feel sorry that I don't have time to interact with family members I sometimes feel as though I am being watched or monitored by the family It is challenging to coordinate or manage remote (virtual) visitations Adapting to the frequent changes in visitation policies is difficult I feel conflicted or guilty about having to enforce visitation rules I find it hard to accept or agree with the current visitation restrictions
Reappraisal of family engagement	Providing information to family members remains important even during visitation restrictions It is still possible to explore ways to engage with families despite restrictions I want to offer families time that helps them feel reassured, even under limited conditions I feel the difficulty of supporting families within a restricted time frame Family visits have a positive impact on patients I have renewed my recognition that family presence serves as emotional support for patients To help patients live in a way that is true to themselves, family cooperation is essential I realised the importance of mental healthcare for families I would like to reflect again on the significance of family nursing
Reflections on policy and practice	I would like to return to practicing family nursing as we did before the COVID‐19 pandemic There is a need to educate and support younger nurses in providing family‐centred care Lessons learned during the pandemic, such as remote visitation, should be carried forward and applied in current practice
Ongoing barriers	I don't have enough time to interact with families due to heavy workload Responding to families is burdensome when nursing duties are demanding It is difficult to predict when families will visit I want to learn more about family nursing and improve my skills Patients' family structures are becoming increasingly diverse There are more patients without reliable or supportive family members Interprofessional collaboration is essential in providing family nursing It is difficult to evaluate family nursing practices effectively In outpatient settings, there are fewer opportunities to engage with families Interacting with diverse families enhances nurses' professional experience

#### 5.4.1 Disruption of Family Nursing

‘Without seeing families face‐to‐face, it was difficult to know the patient's home environment or anticipate their needs after discharge’. The participants described reduced family interaction as a barrier to information sharing, trust building and effective discharge planning. This was especially challenging for early‐career nurses.

#### 5.4.2 Emotional Stress and Moral Conflict

‘I feel sorry that I don't have time to interact with family members’. The participants noted distress from enforcing institutional visitation policies, particularly when they conflicted with their values or clinical judgement.

#### 5.4.3 Reappraisal of Family Engagement

‘Providing information to family members remains important even during visitation restrictions’. Despite these constraints, some participants developed a renewed appreciation for family involvement, noting its emotional and clinical benefits, even when mediated through remote means.

#### 5.4.4 Reflections on Policy and Practice

‘I would like to return to practicing family nursing as we did before the COVID‐19 pandemic’. The participants expressed a desire to restore family interaction to pre‐pandemic levels while incorporating beneficial practices from the pandemic period, such as virtual visits.

#### 5.4.5 Ongoing Barriers

‘I don't have enough time to interact with families due to a heavy workload’. Chronic issues, including time limitations, inconsistent policies and challenging family dynamics remain unresolved. The respondents called for clearer guidelines and professional development to support sustainable family involvement.

## 6 Discussion

This study examined how clinical nurses in Japan perceived and enacted family nursing practice during and after COVID‐19‐related visitation restrictions. Although nurses overwhelmingly acknowledged the theoretical importance of family involvement, the prolonged absence of in‐person contact led to a significant disruption in the systemic relationships that are foundational in family nursing. These disruptions were particularly pronounced among early‐career nurses and those in general ward settings, reflecting that structural constraints rather than attitudinal constraints shaped clinical practice. Framed through family systems theory (Wright and Leahey 2013), these findings underscore the systemic invisibility of families in the clinical setting during the pandemic and the resulting erosion of relational care capacity.

### 6.1 Impact of Visitation Restrictions on Family Nursing Perceptions

This study demonstrated that prolonged family visitation restrictions during the COVID‐19 pandemic have led to significant shifts in nurses' perceptions and implementation of family nursing in Japan. Although most nurses affirmed the theoretical importance of family participation, reintroducing in‐person contact after a prolonged absence revealed new feelings of burden, stress and ambivalence. These findings indicate that family involvement, once inherently valued, may now be perceived as a complex and intrusive aspect of care. This shift may reflect not only individual preferences but also broader structural changes in care routines during the pandemic.

### 6.2 Experience‐Level Differences and Professional Identity

Experience‐based differences were particularly evident. Early career nurses, those who began clinical practice during the pandemic, demonstrated lower affective engagement and greater uncertainty regarding family participation. Framed through Benner's novice to expert model (1984), this may represent an arrested development of relational judgement because these nurses lacked exposure to family interaction as a core component of professional growth. Recent studies have also documented that new graduate nurses during the COVID‐19 pandemic faced significant educational and relational challenges. Casia et al. (2025) reported that the shift from structured bedside instruction to virtual simulation‐based learning left many new nurses feeling confused and less confident in their ability to apply family nursing principles in practice. Similarly, Jerome‐D'Emilia et al. (2022) highlighted that early‐career nurses had limited training in communicating with distressed patients' families and that few institutions provided disaster‐specific education. By contrast, more experienced nurses who practiced in family‐inclusive settings before the pandemic were more likely to integrate families into care and exhibit less resistance to relational responsibilities. This finding suggests that family nursing is a component of professional identity requiring experiential grounding. Taken together, these findings suggest that the pandemic not only limited opportunities for experiential learning but also interrupted the socialisation processes that underpin the formation of professional identity in family nursing.

### 6.3 Relational Care Disruption: A Family Systems Perspective

From the perspective of family systems theory (Wright and Leahey 2013), visitation restrictions caused a rupture in the triadic system of patients, their families and nurses. The nurses reported diminished opportunities to build trust, limited access to contextual family information, and difficulty in coordinating discharge plans. These findings echo previous literature documenting how structural separation undermines care quality and relational continuity (Fukui et al. 2021; Shamali et al. 2023). Particularly in Japan's high‐context communication culture, where unspoken roles and shared understanding are embedded in care, excluding families from bedside practice disrupted nursing care's cultural ecology.

### 6.4 Emotional Burden and Ethical Tensions

The data also revealed significant emotional burdens, including guilt, frustration, and moral distress, especially when nurses were required to implement restrictive visitation policies that conflicted with their internalised care values. These observations align with existing findings on moral distress during COVID‐19 (Carter et al. 2021; Duhamel 2017). From a systems perspective, such distress may reflect a state of role incoherence; nurses were tasked with supporting holistic, family nursing while operating in institutional structures that suppressed those practices. Notably, early career nurses lacking ethical reflection frameworks or role models may have been particularly vulnerable to emotional fatigue and moral confusion during the pandemic.

### 6.5 Implications for Family Nursing Practice and Education

To re‐establish robust family nursing in post‐pandemic practice, we propose several institutional and educational interventions.

- Explicit policies reaffirming the role of families as care partners.
- Simulation‐based training and case‐based reflection to strengthen relational competence. Recent quantitative and intervention studies have demonstrated that simulation‐based education can significantly enhance empathy (Cho and Kim 2024), discussion and reflection skills through long‐term implementation (Nojima et al. 2023), and positive attitudes towards communication skill development among first‐year students (Arrogante et al. 2025). These findings suggest that continuous and structured simulation‐based learning—from undergraduate education to clinical practice—plays a crucial role in developing the communication, discussion, and empathy skills essential for family nursing.
- Designated liaison roles and standardised communication protocols for family engagement.
- Hybrid care models that blend in‐person and virtual interactions for family participation.

Future measurement tools should be considered. This study intentionally prioritised negatively valenced items from the FINC‐NA scale (Benzein et al. 2008) to capture affective discomfort rather than aspirational norms. Items such as ‘I feel that family members check up on me’ revealed significant emotional friction. In contexts where family presence was structurally constrained, scales that assume continuous contact may fail to detect relational gaps or disengagement. Therefore, context‐sensitive subscales that account for the presence and absence of family interactions and their associated affective outcomes should be developed. In culturally layered settings, such as Japan, fostering family nursing requires not only skill development but also conscious efforts to shift the organisational culture that shapes interprofessional and family dynamics.

### 6.6 Limitations

This study had several limitations. First, it relied on self‐administered questionnaires, which may have been influenced by recall or social desirability bias, particularly for emotionally sensitive items related to family presence and professional discomfort. Second, the data were collected from clinical nurses in four general hospitals in Japan, and the findings may not be generalizable to other care settings such as long‐term care, paediatric units, or home‐based care, where family involvement may manifest differently. Third, the cross‐sectional design precluded any inference regarding causality or temporal change, limiting our ability to track attitudinal shifts as visitation policies evolved. Fourth, the study intentionally focused on negatively valenced items from the FINC‐NA scale that were useful for capturing affective frictions but may not reflect the full complexity of nurses' attitudes. Future studies should consider developing mixed‐item or context‐sensitive instruments to assess family nursing attitudes under constrained conditions. Fifth, cultural norms specific to Japan, such as hierarchical relationships, emotional restraint in professional contexts, and institutional conservatism, may have shaped both the responses and broader system‐level dynamics. These cultural factors, while vital for understanding local practices, may limit the direct applicability of the findings to different healthcare systems.

### 6.7 Future Directions

Future research should use longitudinal designs to track how nurses' perceptions of and competencies in family nursing evolve as healthcare systems continue adapting to post‐pandemic realities. Evaluating the effectiveness of targeted interventions, such as structured mentorship programs, ethics‐informed reflective practice, and hybrid communication training that integrates face‐to‐face and virtual modalities, may yield actionable insights into fostering sustainable family‐centred care. Additionally, cross‐national and cross‐setting comparative studies are warranted to unpack the cultural, organisational, and policy‐level variables that shape family nursing practices. This research could contribute to the development of context‐sensitive and globally relevant family nursing models.

## 7 Conclusion

This study emphasised that family nursing cannot be sustained through positive attitudes alone. Family nursing must be structurally supported, relationally experienced, and ethically reinforced. As healthcare systems recover from the pandemic, the reintegration of families into clinical practice must be prioritised, especially for nurses whose formative years of training occurred in isolation from families. Without intentional interventions, a generation of nurses risks internalising an impoverished view of family engagement, not through rejection but through absence.

## Author Contributions

**Makoto Tsukuda:** conceptualization, formal analysis, writing – original draft, writing – review and editing. **Junko Honda:** conceptualization, supervision, writing – review and editing. **Keisuke Nojima:** data collection, formal analysis, writing – review and editing. **Yoshiyasu Ito:** data collection, writing – review and editing. **Hiromi Asada:** data collection, writing – review and editing.

## Funding

This research was supported by a Grant‐in‐Aid for Scientific Research (C) from the Japan Society for the Promotion of Science (JSPS; Grant Number 23K09934).

## Disclosure

Submission with statistics: The authors have checked to make sure that our submission conforms as applicable to the Journal's statistical guidelines described here. There is a statistician on the author team (Keisuke Nojima; email: nojima@tachibana-u.ac.jp). The authors affirm that the methods used in the data analyses are suitably applied to their data within their study design and context, and the statistical findings have been implemented and interpreted correctly. The authors agree to take responsibility for ensuring that the choice of statistical approach is appropriate and is conducted and interpreted correctly as a condition to submit to the Journal.

## Ethics Statement

The study was approved by the Institutional Ethics Review Board of the University of Hyogo (Approval No. 2023F24; Date: 22 November 2023).

## Consent

The voluntary return of the questionnaire constituted the provision of informed consent.

## Conflicts of Interest

The authors declare no conflicts of interest.

## Supporting information


**Appendix S1:** jocn70181‐sup‐0001‐AppendixS1.docx.

## Data Availability

The data that support the findings of this study are available from the corresponding author upon reasonable request.
